# Intrapulmonary administration of bone-marrow derived M1/M2 macrophages to enhance the resolution of LPS-induced lung inflammation: noninvasive monitoring using free-breathing MR and CT imaging protocols

**DOI:** 10.1186/s12880-015-0059-y

**Published:** 2015-05-19

**Authors:** Achraf Al Faraj, Asma Sultana Shaik, Mohammed Alnafea

**Affiliations:** Molecular & Cellular Imaging Lab, Department of Radiological Sciences, College of Applied Medical Sciences, King Saud University, Riyadh, 11433 Saudi Arabia; Prince Naif Health Research Center, College of Medicine, King Saud University, Riyadh, Saudi Arabia

**Keywords:** Lung inflammation, Cell labeling and tracking, Macrophages polarization, Noninvasive pulmonary imaging, Magnetic resonance imaging, Computer tomography

## Abstract

**Background:**

Alveolar macrophages, with their high functional plasticity, were reported to orchestrate the induction and resolution of inflammatory processes in chronic pulmonary diseases. Noninvasive imaging modalities that offer simultaneous monitoring of inflammation progression and tracking of macrophages subpopulations involved in the inflammatory cascade, can provide an ideal and specific diagnostic tool to visualize the action mechanism in its initial stages. Therefore, the purpose of the current study was to evaluate the role of M1 and M2 macrophages in the resolution of lipopolysaccharide (LPS)-induced lung inflammation and monitor this process using noninvasive free-breathing MRI and CT protocols.

**Methods:**

Bone-marrow derived macrophages were first polarized to M1 and M2 macrophages and then labeled with superparamagnetic iron oxide nanoparticles. BALB/c mice with lung inflammation received an intrapulmonary instillation of these *ex vivo* polarized M1 or M2 macrophages. The biodistribution of macrophages subpopulations and the subsequent resolution of lung inflammation were noninvasively monitored using MRI and micro-CT. Confirmatory immunohistochemistry analyses were performed on lung tissue sections using specific macrophage markers.

**Results:**

As expected, large inflammatory areas noninvasively imaged using pulmonary MR and micro-CT were observed within the lungs following LPS challenge. Subsequent intrapulmonary administration of M1 and M2 macrophages resulted in a significant decrease in inflammation starting from 72 h. Confirmatory immunohistochemistry analyses established a progression of lung inflammation with LPS and its subsequent reduction with both macrophages subsets. An enhanced resolution of inflammation was observed with M2 macrophages compared to M1.

**Conclusions:**

The current study demonstrated that *ex vivo* polarized macrophages decreased LPS-induced lung inflammation. Noninvasive free-breathing MR and CT imaging protocols enabled efficient monitoring of progression and resolution of lung inflammation.

## Background

Apart from their well-known role in phagocytosis and recognition of foreign antigens, alveolar macrophages (AM) play a key role in the induction and the resolution of inflammatory responses characteristic of lung diseases such as chronic obstructive pulmonary diseases (COPD) and acute lung inflammation (ALI) [1, 2]. The termination and the resolution of lung inflammation and the consequent tissue homeostasis are remarkably functional and coordinated processes orchestrated by AM [3, 4]. Although the diversity of macrophage function is not yet fully understood, it is increasingly recognized that AM are endowed with high functional plasticity allowing them to acquire either a classical pro-inflammatory M1 activation (M1 macrophages) or an alternatively immunoregulatory M2 activation (M2 macrophages) depending on the cross-talk of various signals they received from surrounding cells or from the pathogen itself [5, 6]. While M1 macrophages are endowed with strong tumoricidal and microbicidal activity, M2 macrophages are implicated in containment and promotion of tissue remodeling and disease progression [7, 8]. Taken together, macrophages populations perform homeostatic activities such as host defense, wound healing and immune regulation [9].

Thus, polarized macrophages subpopulations may considerably help in both diagnostic purposes and evaluation of therapeutic interventions through their inherent ability to home at the inflammatory sites making them attractive vehicles to deliver contrast agents [10]. Consequently, noninvasive imaging approaches, which enable simultaneous monitoring of inflammation progression and tracking of a particular cell population involved in the inflammatory cascade, can provide an ideal and specific diagnostic tool to visualize the action mechanism in its initial stages.

Macrophage imaging using magnetic resonance imaging (MRI) has emerged as a promising noninvasive tool for pre-clinical and clinical assessments of several inflammatory diseases such as atherosclerosis and myocardial infarction, stroke, multiple sclerosis, rheumatoid arthritis, and kidney transplantation [11]. The homing of different macrophages subsets to the inflammatory sites has been previously demonstrated using high resolution noninvasive MRI in a mice model bearing calf muscle inflammation [12].

Noninvasive monitoring of macrophages trafficking to the lung using MRI was challenging because of the difficulties to image this organ (i.e. signal loss due to cardiac pulsation and respiration, susceptibility artifacts caused by multiple air-tissue interfaces and low proton density). However, the technical improvements in the gradient systems and the development of ultrashort time of echo (UTE) MR pulse sequence [13] have made possible attaining both high spatial resolution and good signal to noise ratio in lungs MR images [14]. Besides, pulmonary inflammatory diseases involving edema formation or mucus production as observed in COPD, Asthma or ALI [15], can be directly measured using standard gradient echo sequences [16] and with a higher sensitivity and diagnostic usefulness using UTE in artefacts-free images. Therefore, simultaneous noninvasive tracking of macrophages biodistribution and monitoring of inflammation progression using MRI will certainly open new perspectives for imaging, diagnosis and treatment of several respiratory diseases.

On the other hand, with the enhanced improvements in computer speed and memory and the inherent X-ray absorption contrast between lung parenchyma and gaseous exchange, micro computed tomography (micro-CT) has in recent time become a powerful noninvasive imaging technique. Micro-CT has the advantage of offering the highest spatial resolution among the noninvasive imaging modalities thus providing information about lung function and changing tissue characteristics in a free-breathing rapid acquisition protocol during longitudinal follow-up studies [17]. It also allowed evaluating acute and chronic pulmonary disease models to understand lung parenchyma and the bronchial tree both structurally and functionally [18, 19].

In the current study, the effect of intrapulmonary administration of bone marrow derived macrophage (BMDM) subpopulations to decrease LPS-induced lung inflammation was evaluated in a mouse model using noninvasive free-breathing high-resolution MRI and CT protocols. Histological analyses were performed to correlate and confirm the noninvasive imaging readouts.

## Materials and methods

### Animals and COPD model

Female Balb/c mice (20-22 g) were obtained from the University’s main animal care center. All experiments were performed in accordance with the National guidelines for the care of laboratory animals and the study was approved by the Ethical Committee of the College of Applied Medical Sciences (agreement number: CAMS05/3334). During the different experimental procedures, animals were anesthetized by intramuscular administration of a mixture of 0.1 mL of 4 mL of ketamine (50 mg/mL), 1 mL of xylazine (2 %), and 5 mL of physiological serum. COPD model was induced by intrapulmonary instillation (1 mg.kg^−1^; V = 100 μl) of Lipopolysaccharide (LPS) from Escherichia Coli (Santa Cruz Biotechnology, Inc., CA, USA) using a MicroSprayer aerosolizer (Penn-Century Inc., PA, USA). At 48 h post LPS challenge, mice were intrapulmonary instilled with either physiological saline, iron labeled *ex vivo* polarized M1 or M2 macrophages (10^6^ cells suspended in 100 μl phosphate buffered saline (PBS) solution).

### Macrophages polarization and magnetic labeling

Bone marrow (BM) derived M1 and M2 macrophages were obtained and polarized as previously reported [12]. Briefly, BM cells from tibiae and femora of donor mice were incubated for 7 days at 37 °C in complete IMDM medium containing L-glutamine and phenol red (Gibco, Lifetechnologies, CA, USA) and supplemented with 10 ng/ml of macrophage clone stimulating factor (R&D systems, Abingdon, UK) to obtain adherent M0 macrophages. Macrophage polarization was then induced by incubating adherent M0 cells for 20 h at 37 °C in complete IMDM medium supplemented with 1 ng.ml^−1^ LPS (Santa Cruz Biotechnology, Inc., CA, USA) and 10 ng.ml^−1^ INFγ to obtain M1-polarized cells or with 10 ng.ml^−1^ IL-10 and 20 ng.ml^−1^ IL-4 (R&D systems, Abingdon, UK) to obtain M2-polarized macrophages.

M1 or M2 macrophages were labeled with Dextran-coated SPIO nanoparticles (Micromod Partikeltechnologie GmbH, Germany) at an extracellular iron concentration of 2 mM with 1 h incubation time at 37 °C, which was chosen as the best compromise between labeling efficiency and biocompatibility to the cells. For efficient macrophages labeling, SPIO nanoparticles were functionalized by the addition of polyethylene glycol (PEG) with amine terminal (NH_2_) [20]. We have previously reported that these nanoparticles showed an enhanced labeling efficiency of macrophages with a better biocompatibility [21].

Macrophages were incubated in serum-free RPMI medium containing L-glutamine and phenol red (Gibco, Lifetechnologies, CA, USA) to avoid proteins interactions with the uptake mechanism [22]. The incubation step was followed by an overnight chase period in SPIO-free culture medium to allow sufficient time for iron oxide internalization. Prior to administration, iron content in the different macrophages subsets were quantified using Ferrozine-based assay and their biocompatibility was assessed using MTT assay as previously reported [21].

Ferrozine is an iron-chelating agent that forms a complex with iron and exhibits characteristic UV/Vis absorption. By comparing to a standard calibration curve, accurate quantification of iron uptake by macrophages can be obtained. Briefly, 2x10^5^ labeled M1 or M2 macrophages were digested with 5 M hydrochloric acid and absorbance was measured at 351 nm using Multiskan Go Spectrophotometer (Thermo Scientific, NH, USA). Using a calibration curve of the absorbance vs. iron concentration of the SPIO nanoparticles prepared under the same experimental protocol, the iron content in the macrophages was determined and expressed as pg of iron per cell.

Cell viability was evaluated by MTT (3-[4,5-dimethylthiazol-2-yl]-2,5- diphenyltetrazolium bromide) Cell Growth Assay Kit (Merck Millipore, MA, USA) according to the manufacturer protocol. Briefly, iron labeled M1 and M2 macrophages (10^4^ cells/well) were placed in a 96-well plate (n = 3) and absorbance was measured using Multiskan Go Microplate Spectrophotometer (Thermo Scientific, NH, USA) with a test wavelength of 570 nm and a reference wavelength of 630 nm. The relative percentage of cell viability for each condition was calculated related to unlabeled M1 and M2 macrophages.

#### Magnetic resonance imaging

To noninvasively monitor the biodistribution of M1 or M2 iron-labeled macrophages subpopulations in LPS-induced pulmonary model, mice were imaged using a 4.7 T Pharmascan 47/16 Bruker magnet interfaced to ParaVision 5.1 software (Bruker Biospin GmbH, Rheinstetten, Germany). A free-breathing MR imaging protocol was optimized to allow simultaneous detection of macrophages subsets and the visualization of inflammation progression in the lung using a radial UTE sequence (TR/TE = 100/0.4 ms) with 100 x 100 μm pixel resolution according to the protocol previously reported [23]. Pulmonary MRI was performed on mice (n = 6 per group) before LPS challenge (Control), 48 h post LPS challenge (LPS) chosen as time 0 for investigating the effect of macrophages intrapulmonary administration on the resolution of lung inflammation, and at 4 h, 24 h, 48 h, 72 h and 168 h after either iron-labeled M1 or M2 macrophages subsets administration (LPS + M1 or LPS + M2, respectively). A set of 12 consecutive axial slices with 1 mm thickness were positioned approximately at the same level for all the animals in order to cover the whole lung volume during the 1-week follow-up study.

MR images were analyzed with freeware medical image analysis software (MIPAV, National Institutes of Health, Bethesda, MD, USA). The inflamed lung volume (ILV) was quantified with a semiautomatic segmentation procedure [14]. Briefly, a low-intensity threshold was applied to exclude the non-inflamed regions from the lung images. LPS-induced inflammatory regions were extracted with a semiautomatic tool capable of generating contours whilst moving over the different lung structures by recognizing the intensity level of each pixel and selecting regions according to the default preset parameters (MIPAV ‘LevelSet’ active-contour algorithm). The total volume of high-intensity signals was computed by multiplying the slice thickness by the sum of all the areas segmented in the 12 consecutive slices. The segmentation parameters were the same for all the analyzed images, chosen to segment regions corresponding to high-intensity signals. As the signals from the LPS-induced edema and the vessels were of comparable intensities, the volume corresponding to the vessels was assessed on control images and then subtracted from the volumes determined on post-challenge images.

#### Computed tomography imaging

To monitor using an alternative noninvasive imaging modality the progression of LPS-induced inflammation and assess the possibility of inflammation resolution after intrapulmonary administration of either M1 or M2 macrophages subpopulations, mice (n = 6 per group) were scanned using a dedicated small animal high resolution micro-CT scanner (SkyScan 1176, Kontich, Belgium). The following parameters were used: 50 kV, 1 mm Al filter, 385 μA source current, 600 ms exposure time. Micro-CT imaging was only performed at 24 h, 72 h and 168 h post-macrophages administration on the same mice, which were previously imaged using MRI. Mice were allowed to recover for 4 to 6 h after the MRI acquisitions. CT scans were limited to three time points during the 1-week investigation to avoid high radiation exposure to the mice (i.e. ~ 0.1Gy per scan) that were imaged at the different investigation time points. Projection images were recorded in steps of 0.5 degrees from 0 to 360 degrees. Images were acquired throughout the spontaneous respiratory cycle. Respiratory motions were recorded with a visual camera, detecting the up- and downward movement of the thorax and then translated into a pseudo-sinusoidal signal to allow retrospective respiratory gating. 3D acquisitions with a 18 μm isotropic resolution were performed to cover the entire lung for a total acquisition time of 24 min. Images were reconstructed with SkyScan NRecon software (version 1.6.9.4) using a Feldkamp cone-beam reconstruction algorithm. Reconstruction parameters were smoothing “6”, beam-hardening correction “22 %”; post-alignment and ring artifact correction were optimally set for each individual scan. Reconstructed 3D images have a total of 864 slices with isotropic 18 μm voxel size and 1024 × 1024 resolution. A water phantom was used to calibrate the image to Hounsfield units (HU). Lungs were automatically delineated in the 3D micro-CT images as previously described [24] and the airways were removed from the lung delineation [25]. Inflamed lung volume (ILV) was finally measured on the segmented lungs to quantify the level of LPS-induced inflammation detected using CT and subtracted from values quantified in control mice to exclude the volume corresponding to the vessels and correlate with MRI readouts.

#### Immunohistochemistry

Lungs (n = 3 in each group and at each time point) were directly removed after the micro-CT scanning and fixed overnight in 4 % paraformaldehyde. Processing of tissues for histological analyses was performed on sets of consecutive 5 μm thick sections. F4/80 rat monoclonal antibody (1:100) as universal marker for macrophages, NOS2 rabbit polyclonal antibody (1:1000) and Arginase goat polyclonal antibody (1:100) (Santa Cruz Biotechnology, Inc., CA, USA) as marker for M1 and M2 macrophages respectively, were applied as primary antibodies. Respective mouse ABC staining systems were used as sources for secondary antibodies (Santa Cruz Biotechnology, Inc., CA, USA) and consequent detection was performed as per manufacturer’s instructions. Slides were counterstained with hematoxylin before being observed using a BX53 Olympus microscope and analyzed using CellSens Entry digital imaging software (Olympus Corporation, Tokyo, Japan).

#### Statistical analysis

Data were presented as the mean standard deviation. ANOVA with post-hoc analyses were performed using SPSS software v12.0 (SPSS Inc., Chicago, IL, USA) to compare ILV assessments among the different groups. A p-value < 0.05 was considered significant for all tests.

## Results

Prior to their *in vivo* administration, M1 and M2 labeling efficiency and viability were evaluated. Quantification of iron content revealed an uptake of 17.68 ± 0.95 and 21.12 ± 1.25 pg/cell for M1 and M2 macrophages, respectively, under the used experimental conditions, while showing 99.72 ± 2.5 % and 99.38 ± 1.6 % of relative cell viability.

**Fig. 1:**
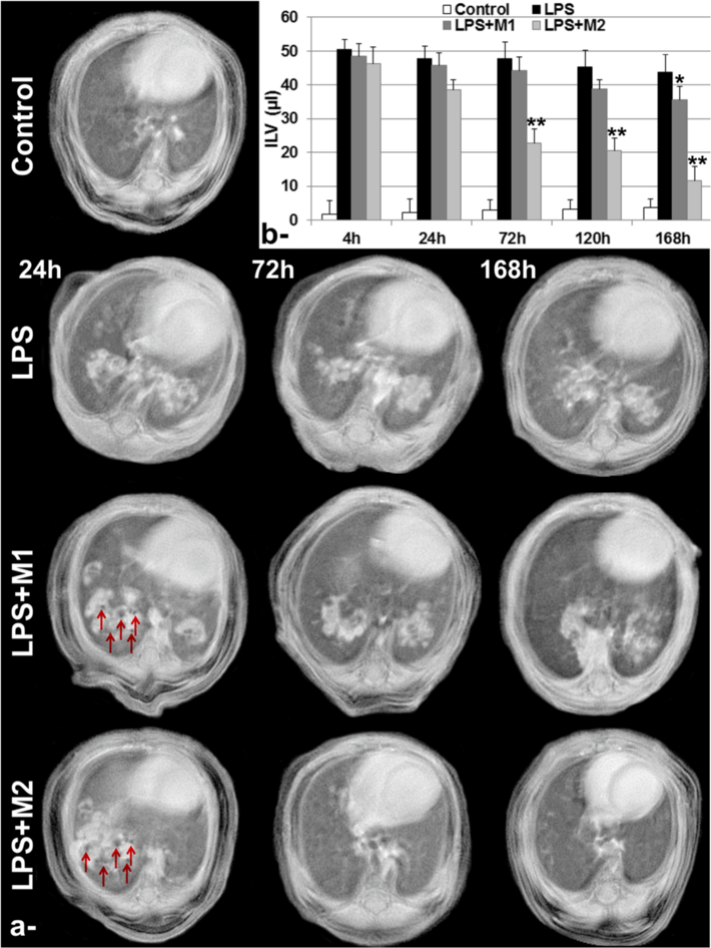
Lung Magnetic Resonance Imaging. **a**- Representative axial MR images of Control, LPS, LPS + M and LPS + M2 groups at 24 h, 72 h and 168 h post intrapulmonary administration of either M1 or M2 bone-marrow derived macrophages subpopulations. Red arrows highlight the presence of void signal dots, related to the presence of iron-labeled macrophages co-localized with inflammatory regions in the lung. **b**- Quantification of inflamed lung volume (ILV) for the different groups during the 1-week follow-up investigation. Data expressed as mean ± SD, *n* = 6 per group. * *p* < 0.05. **p < 0.01

Pulmonary MR images acquired using UTE sequence, at 48 h post LPS challenge with 1 mg.kg^−1^, allowed the detection of large inflammatory areas in the lung (Fig. 1a). At this time point, the average inflamed lung volume measured in the LPS group was 50.4 ± 3.7 μl and found to decrease slightly during the 1-week investigation to reach 43.8 ± 5.3 μl, whereas the average quantified value of ILV was 1.8 ± 3.9 μl in the control group (Fig. 1b). Following intrapulmonary administration of either M1 or M2 macrophages, void signal dots, related to the presence of iron-labeled macrophages were detected in the lung (red arrows in Fig. 1a) at 24 h post administration. Interestingly, a statistically significant decrease in inflammation was detected starting from 72 h post intrapulmonary administration of M2 macrophages with ILV equal to 22.8 ± 4.1 μl and 11.6 ± 4.2 μl at 72 h and 168 h, respectively, compared to 47.8 ± 4.9 μl for LPS group before M2 macrophages administration. However, a lower effect was observed with M1 macrophages subsets, which was statistically different at 168 h post administration. At this time point, ILV decreased from 43.8 ± 5.3 μl for LPS group to 35.6 ± 3.9 μl after M1 macrophages administration.

**Fig. 2:**
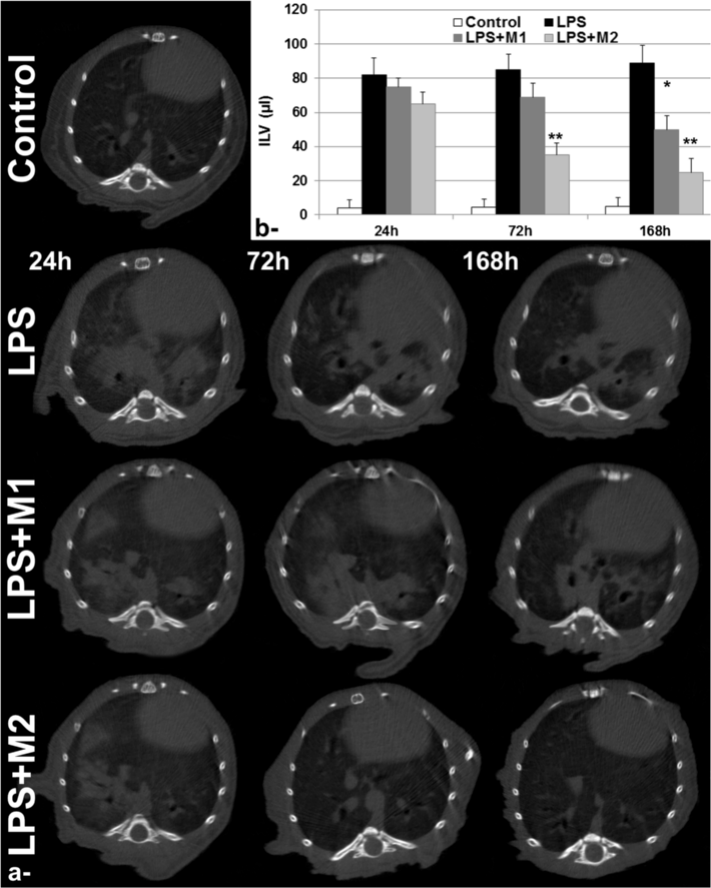
Lung Computed Tomography imaging. **a**- Representative axial micro-CT images of Control, LPS, LPS + M1 and LPS + M2 groups at 24 h, 72 h and 168 h post intrapulmonary administration of either M1 or M2 bone-marrow derived macrophages subpopulations. **b**- Quantification of inflamed lung volume (ILV) for the different groups during the 1-week follow-up investigation. Data expressed as mean ± SD, *n* = 6 per group. * *p* < 0.05. **p < 0.01

Similarly, micro-CT images acquired at 24 h post LPS challenge allowed the detection of inflammation with ILV = 82.1 μl in LPS group compared to ILV = 3.9 μl in Control group, as assessed using CT quantification, (Fig. 2). Following macrophages administration, considerable attenuation in inflammation was observed with M2 macrophages subsets starting from 72 h post intrapulmonary administration corresponding to 41.2 % decrease in ILV compared with LPS group at the same time point. M1 macrophages subsets were found to produce a statistically significant attenuation in ILV at 1-week investigation time point. CT did not allowed direct visualization or detection of macrophages subpopulations even with its very high 3D isotopic resolution of 18 μm.

**Fig. 3:**
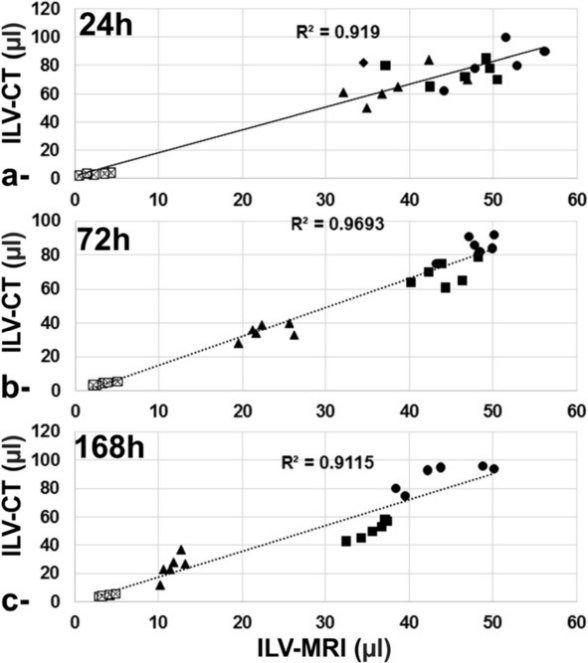
MRI vs. CT correlation. Correlation plot of the inflamed lung volume (ILV) quantified using both MRI and CT at **a**- 24 h; **b**- 72 h and **c**-168 h investigation time points. ⌧: control, ●: LPS, ■: LPS + M1, ▲: LPS + M2

An excellent correlation was found between the quantification of ILV assessed using either MRI or CT with a correlation coefficient of 0.919, 0.9693 and 0.9115 at 24 h, 72 h and 168 h investigation time points, respectively (Fig. 3).

**Fig. 4:**
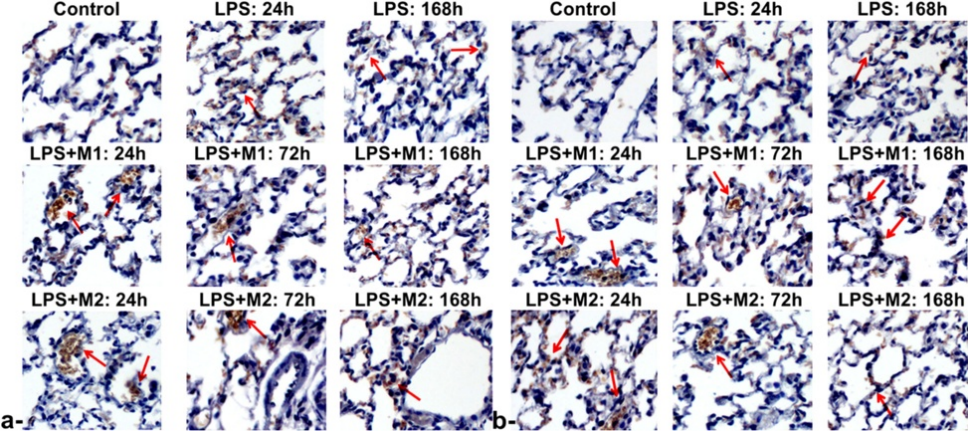
Immunohistochemistry (IHC) analyse. Representative lung images of Control, LPS, LPS + M1 and LPS + M2 at 24 h, 72 h and 168 h post-intrapulmonary administration of either M1 or M2 bone-marrow derived macrophages subpopulations **a**- NOS2 **b**- Arginase1 staining

To investigate the effect of intrapulmonary administration of *ex vivo* polarized macrophages subtypes on the attenuation of lung inflammation, immunohistochemistry analyses were also performed to confirm noninvasive imaging readouts at 24 h, 72 h and 168 h post treatment with LPS, LPS + M1 and LPS + M2 compared to Control mice. IHC analyses (Fig. 4) revealed strong accumulation of inflammatory cells, changes in alveolar walls and airway narrowing. While LPS induced considerable inflammation in the lungs over time compared to controls, administration of macrophages seems to have counteracted the inflammation by helping the lungs revert to their normal histology. The intrapulmonary administration of either M1 or M2 macrophages revealed an attenuation of inflammation with a more prominent effect observed following instillation of M2 macrophages (i.e., Arginase1 marker) compared to mice receiving M1 macrophages (i.e., NOS2 marker). Considerable attenuation of lung inflammation was evident histologically post macrophages instillation.

## Discussion

This study evaluated the possibility of enhanced resolution in LPS-induced lung inflammation following intrapulmonary administration of *ex vivo* polarized M1 and M2 bone marrow derived macrophages. Free-breathing noninvasive MR and CT imaging acquisitions along with confirmatory histological analyses were used to monitor this process.

We have recently demonstrated the possibility of noninvasive tracking the preferential migration of differently polarized macrophages after their intravenous injection to the sites of inflammation in the lung [23]. In the current study, following their intrapulmonary instillation, iron labeled M1 and M2 macrophages subsets were successfully detected in MR images as void signal dots up to 24 h post administration. Macrophages were found to co-localize with the inflammatory regions simultaneously detected as hyper-intensity signal in artifact-free images using UTE sequence. Void signal dots were not perceived at later investigation time point, most probably because of the biodegradation of iron oxide nanoparticles by the macrophages. It was previously reported that the T1 and T2 relaxivities of iron oxide nanoparticles, which allow their noninvasive detection using MRI, decreased following internalization into macrophages and their subsequent degradation [26].

On the other hand, LPS-induced lung inflammation, which was reported to peak at 48 h post LPS challenge and manifested in edema secretion resulting from generalized granulocytic (especially neutrophilic) inflammation [27], was chosen as the time point to investigate the enhanced resolution of lung inflammation following macrophages intrapulmonary administration.

Inflamed lung volume, reported using both MRI and CT, revealed a considerable increase in inflammation following LPS treatment. An enhanced resolution of pulmonary inflammation quantified by a statistically significant decrease in ILV was observed following administration of M2 macrophages starting from 72 h post administration (i.e. 52.3 % to 73.5 % decrease as assessed by MRI at 72 h and 168 h, respectively) and to a lower extent with M1 macrophages 1-week post administration (i.e. 18.7 % decrease). With its higher spatial resolution, ILV assessed by CT was found higher as compared with MRI readouts. However, quantification results obtained using both noninvasive imaging modalities, performed under free-breathing protocol, were perfectly correlating.

Corroborating the findings of MRI and CT, we detected through histology the successful buildup of externally administered macrophages through demonstration of F4/80, a universal marker for these cells (data not shown). In addition, successful homing of both M1 and M2 macrophages subsets was demonstrated through the identification of iNOS and Arginase1 markers, respectively. Architectural changes in the lungs have been clearly demonstrated with LPS along with enhanced recruitment of macrophages [28]. Lung inflammation was found to decrease gradually in a time dependent manner following intrapulmonary administration of macrophages subsets with a statistically significant attenuation observed as early as 72 h in LPS + M2 group (p < 0.01) and by 7 days in LPS + M1 group (p < 0.05). These observations confirmed the potential role of *ex vivo* polarized bone marrow derived macrophages subsets in the attenuation of inflammation. In addition, it is important to note that the presence of iron oxide labeled macrophages after instillation has been confirmed in lung histology sections and the results were published elsewhere [23, 29].

Studies have reported that macrophage polarization is based on the levels of various cell signals which drive macrophages to continuously switch from M1-proinflammatory to M2-immunomodulating functions and vice versa to help in regulating and resolving inflammation [1–3, 6]. While the process involving the initiation and amplification of the inflammatory responses were extensively studied, the mechanisms favoring the resolution of lung inflammation is not completely understood. Different mechanisms were proposed for the suppression of LPS-induced inflammation. Lonescu et al. [30] suggested that soluble factors secreted by mesenchymal stem cells may promote the resolution of lung injury in part by modulating alveolar macrophage function. Do-Umehara et al. [31] reported that the transcription factor Miz1 was involved in the termination of LPS-induced inflammation. D’Alessio et al. [32] suggested that monocyte-derived iNOS plays a pivotal role in mediating resolution of inflammation by modulating lung immune responses, thus facilitating the clearance of alveolar inflammation and promoting lung repair. A more recent study by Mauer et al. reported that IL-6 production by M1 macrophages might lead to a switch from M1 to M2 macrophages in order to resolve the inflammation [33].

## Conclusions

In conclusion, *ex vivo* polarized M2 bone marrow derived macrophages were found to enhance the resolution of LPS-induced lung inflammation. Noninvasive imaging modalities used in the current study can have the advantage of performing longitudinal follow-up studies on disease progression and providing detailed information on the location and extension of the pathology. The study findings may open new therapeutic options by using bone marrow derived macrophages for reducing tissue inflammation. However, the potential cell signals secreted by macrophages and their mechanism of action in target tissue sites need further evaluation in both pre-clinical and clinical settings on a larger scale.
